# Particle Detection in Free-Falling Nanoliter Droplets

**DOI:** 10.3390/mi15060735

**Published:** 2024-05-31

**Authors:** Fabian Sturm, Viktoria Zieger, Peter Koltay, Daniel Frejek, Sabrina Kartmann

**Affiliations:** 1 Laboratory for MEMS Applications, IMTEK—Department of Microsystems Engineering, University of Freiburg, 79110 Freiburg, Germany; 2 Hahn-Schickard, 79110 Freiburg, Germany

**Keywords:** optical particle detection, spheroid detection, nanoliter dispensing, drop-on-demand, glare point illumination, bioprinting

## Abstract

Sorting and dispensing distinct numbers of cellular aggregates enables the creation of three-dimensional (3D) in vitro models that replicate in vivo tissues, such as tumor tissue, with realistic metabolic properties. One method for creating these models involves utilizing Drop-on-Demand (DoD) dispensing of individual Multicellular Spheroids (MCSs) according to material jetting processes. In the DoD approach, a droplet dispenser ejects droplets containing these MCSs. For the reliable printing of tissue models, the exact number of dispensed MCSs must be determined. Current systems are designed to detect MCSs in the nozzle region prior to the dispensing process. However, due to surface effects, in some cases the spheroids that are detected adhere to the nozzle and are not dispensed with the droplet as expected. In contrast, detection that is carried out only after the droplet has been ejected is not affected by this issue. This work presents a system that can detect micrometer-sized synthetic or biological particles within free-falling droplets with a volume of about 30 nanoliters. Different illumination modalities and detection algorithms were tested. For a glare point projection-based approach, detection accuracies of an average of 95% were achieved for polymer particles and MCF-7 spheroids with diameters above 75 μm. For smaller particles the detection accuracy was still in the range of 70%. An approach with diffuse white light illumination demonstrated an improvement for the detection of small opaque particles. Accuracies up to 96% were achieved using this concept. This makes the two demonstrated methods suitable for improving the accuracy and quality control of particle detection in droplets for Drop-on-Demand techniques and for bioprinting.

## 1. Introduction

In recent times, material jetting processes have gained increasing importance, particularly in the field of three-dimensional bioprinting and bioassembly [1,2,3,4,5,6].

Cellular aggregates, such as spheroids and organoids, can serve as building blocks for organ-printing processes by dispensing distinct amounts of these aggregates into hydrogel structures [7]. Furthermore, the three-dimensional structure of spheroids and organoids can be used to replicate metabolic tissue properties in a realistic manner, thus enabling the testing of their reaction against drugs [2,3,4,5,6]. In all of the previously mentioned cases, distinct amounts of cellular aggregates must be deposited, which is predominantly achieved through material jetting processes [2,4,5,6].

The process of material jetting describes the deposition of build materials by forming droplets and depositing these droplets on distinct target spots [8]. The generation of droplets is typically achieved through the use of piezoelectric or thermal ink jetting [1,2,4,8]. Hereby, thermal expansion or piezoelectric displacement of a plunger generates displacement pressure pulses which eject droplets [5,8]. The integration of automatic sorting and selection of individual cellular aggregates, such as spheroids, contained within the build material facilitates the handling of these systems, thereby increasing throughput and significantly reducing processing times [1,2,4]. The aforementioned process is alternatively referred to as the Drop-on-Demand approach, as described in [2,4].

For the deposition of distinct amounts of cellular aggregates, reliable detection of dispensed aggregates is required. In most cases, a label-free approach for the detection of theses cellular aggregates is desired, as labels can influence aggregates and sequential experiments carried out on these aggregates [1,2,3,4,6]. To ensure a label-free detection approach, the number of dispensed spheroids is usually tracked by monitoring the nozzle region of the dispenser, predicting the amount of aggregates dispensed with the next droplet [2,3,4,7]. The systems of Zieger et al., as well as the system of Parent et al., demonstrated high detection accuracies within the capillary for spheroids in the size range of 50 μm up to 200 μm [4,7]. However, these methods depend on the optical properties of the observed nozzle region or dispense head, which limits the possibilities of applied dispensers [4,6]. Additionally, the opaqueness of the meniscus region of the dispensing nozzle can obscure a particle, hence making impossible the detection of whether the particle has been dispensed or is hidden in the meniscus [3,4]. Therefore, we propose the detection of cellular aggregates in already ejected, free-falling droplets, enhancing the independence of the detection process from dispense head geometry and materials.

So far, further solutions for characterization of free-falling droplets have mainly focused on the size and velocity of the droplets [9,10]. Due to the spherical lens behavior of droplets, which leads to the focusing of incident light towards the center of the droplet, imaging of the contained substances within the droplets becomes an issue [11,12]. Resulting images of droplets that are illuminated by only one light source show dark edges due to the focusing of the light [2,12].

Approaches to detect single cells as small as 25 μm within droplets by characterizing the velocity or deceleration of droplets have already been demonstrated by Huang et al. [13]. By applying a machine learning algorithm to the generated data, detection accuracies between 77% and 82% were achieved [13]. Nevertheless, real-time detection of particles within the droplets is not feasible using this concept due to the high computational effort required. Furthermore, the detection accuracies are considerably inferior to those of the concepts which detect particles within the capillary [4,7,13].

In contrast to direct imaging approaches and the characterization of droplets, Heinisch investigated content-dependent glare point changes on the droplet surface [14]. With that, they were able to detect the position and size of enclosed air bubbles in trapped droplets. Here, our goal is to use glare point changes on the image of the free-falling droplet to detect enclosed spheroids for a more precise and stable determination of the number of spheroids dispensed.

To optically detect particles inside a spherical object, it is necessary to understand the propagation of light through a spherical object.

**Figure 1:**
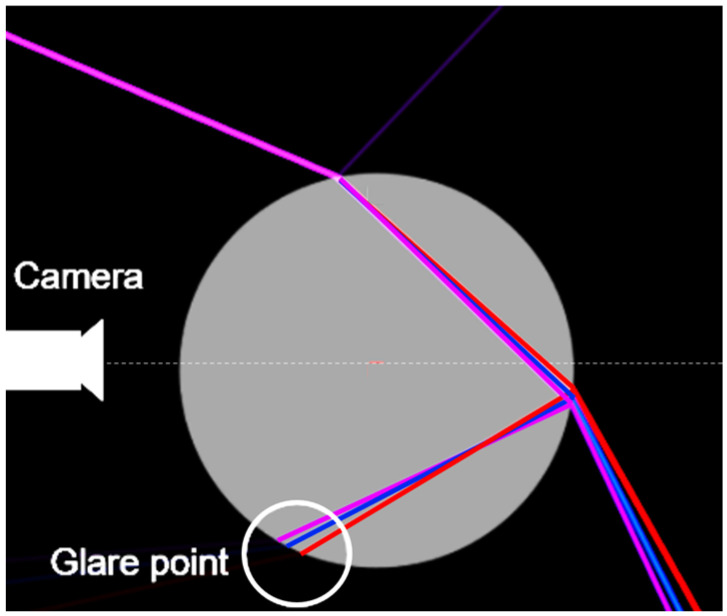
Simulation of the ray propagation of different wavelength (405 nm, 465 nm, and 635 nm) hitting a droplet, demonstrated by the gray circle (refractive index of water 1.33 was applied), at an angle of approx. 40°. The difference in reflection angles due to the different wavelengths results in the slightly shifted position of the glare points (white circle) depending on the wavelength, after being reflected on the inside of the droplet [16].

Light-scattering properties on droplets or translucent spherical particles are described by the Gustav Mie theory [15]. For spherical particles without an inclusion, this theory can be easily applied and is already the basis of many droplet- and particle-based measurement techniques [14,15]. In contrast, for spherical objects or droplets with an inclusion, such as air bubbles or cellular aggregates, this theory must be extended to a much more complex form, which leads to increased computational effort [14,15]. To investigate light propagation for the illumination concepts in this work, we do not follow this approach for these reasons. Instead, the ray optic theory is sufficient for a basic understanding of light propagation. The results of this approach for illuminating a droplet with different wavelengths can be seen in Figure 1.

From the perspective of geometrical ray optics, the surface of the droplet causes refraction of the incident beam that strikes the droplet surface. The ray enters a medium with a higher refractive index than the ambient medium, causing refraction towards the centre of the droplet. Subsequently, the light is partially reflected at the phase transitions from an optically denser medium to an optically thinner medium (ambient air) on the back of the droplet. Total reflection can be achieved, contingent on the medium of the droplet and the angle of incidence.

The reflected light beam is directed back towards the front of the droplet. This phenomenon allows the observation of characteristic glare points for empty droplets, which are dependent on the aforementioned variables.

Enclosed particles cause changes in the described light propagation due to absorption and scattering effects [14]. For cellular aggregates like spheroids, organoids, or microtissue, light penetration is limited by the different nonhomogeneous refractive index distributions which result from the different components of the cells. Changes in the speed and angle of light propagation are induced and promote the scattering and absorption of the light [17].

These alterations in light propagation, which are contingent upon the dimensions, composition, and scattering characteristics of the particles within the droplet, are anticipated to manifest as the emergence or absence of glare points or alterations in the dimensions and brightness of the preexisting glare points on the droplet surface. However, this phenomenon occurs only when the light interacts with the particle during its propagation through the droplet. The use of specially designed droplet illuminations, which either project glare points onto the droplet surface or reduce the optical effects caused by the lens behavior of the droplet due to light entering the droplet in a diffuse way from every direction, will be employed in the following section to investigate the efficacy of different approaches to particle detection in free-falling droplets.

## 2. Materials and Methods

### 2.1. Spheroid Culture

Spheroids are three-dimensional cell culture aggregates that can mimic tissue as well as micro tumors much better than two-dimensional cell culture models can. They provide strong cell-to-cell bonds, and the three-dimensional structure realistically mimics the resulting drug diffusion barrier. This makes spheroids especially interesting for drug and cancer research [18].

For this study, MCF-7 breast cancer cells were used to generate spheroids. They were generated by means of the hanging drop method [1] or the use of Corning^®^ Elplasia^®^ 12K Flasks (Corning, New York, NY, USA). The seeding concentration was selected in accordance with the manufacturer’s instructions to generate spheroids with a diameter between 80 and 130 micrometers.

### 2.2. Imaging Setup

In order to replicate the functionality of a Drop-on-Demand material jetting bioprinting device, a non-contact nanodispenser (PipeJet Nanodispenser, BioFluidiX, Breisgau, Germany) was employed. The device is equipped with a deformable polyimide capillary (Zeus Inc., Orangeburg, SC, USA) with an internal diameter of 250 μm. The length of the capillary is 200 mm. One end of the capillary is fluidically coupled with a reservoir (Eppendorf 1.5 mL tubes, Thermo Fisher Scientific, Waltham, WA, USA) containing different test liquids, including suspended particles or spheroids. Capillary forces draw liquid through the capillary.

The opposite end of the capillary is connected to the PipeJet module, where it functions as a nozzle. As the nanodispenser compresses the capillary, the droplets are ejected, displacing the liquid particle suspension within the capillary. The compression of the capillary is achieved by a piezoelectric plunger, as previously demonstrated by Streule et al. [19]. As a result of this displacement, particles are randomly aspirated into the capillary from the reservoir and transported through the capillary to the dispensing nozzle region, where they are eventually ejected within the dispensed droplet. By regulating the spheroid concentration in the reservoir, the spheroid throughput through the capillary can be varied (see Zieger et al., 2022 [2]). A concentration of less than 300 spheroids per milliliter was selected in order to minimize the risk of dispensing multiple spheroids within a single droplet. Nevertheless, the procedure must be capable of accommodating a considerable number of empty droplets. Provided that droplets containing particles are ejected intermittently, the aforementioned disadvantage does not have a detrimental impact on the experiments. It is essential that droplet size is consistent in order to ensure the reproducibility of the results, which will be solely dependent on the droplet content. In the experiments performed by Streule et al., it was proven that the droplets ejected by the nanodispenser and capillary setup are similar in shape and velocity, with a standard deviation of the dosage volume of only 1.6% [19].

**Figure 2:**
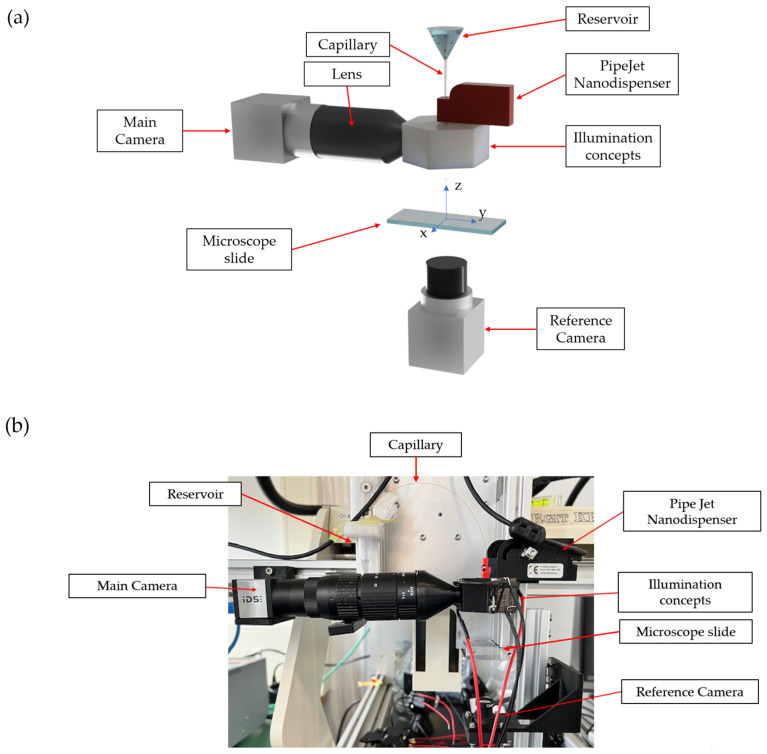
Test setup with all components that were used to perform the experiments. (**a**) Schematic overview of the components. (**b**) Physical setup built in the laboratory.

The volume of the droplets is approximately 30 nanoliters. Subsequent to the dispensing event, the droplet passes the illumination and imaging module in a free-falling trajectory through the air, without physical contact with other components. A CMOS camera with a resolution of 1280 × 1024 pixels (IDS UI-3240CP-M-GL Rev.2, IDS Imaging Development GmbH, Obersulm, Germany) is employed to capture images of the droplets. Furthermore, a Computar Macrozoom MLM-3XMP lens (CBC Group, CapitaSpring, Singapore) is employed to enhance the magnification of the droplet in the image. Additionally, lenses with a higher magnification were also tested. However, the lenses with lower magnification proved to be sufficient for the observation of changes in the glare points, particularly in the case of the tested sizes of spheroids. The application of a higher magnification did not result in the acquisition of any additional information regarding the particles under investigation. Consequently, the time required for image processing was extended due to the magnified droplet within the image, resulting in a greater number of pixels that needed to be analyzed. The complete setup, including the selected lens, is illustrated in Figure 2. The magnification range of the chosen computer lens was 0.3× to 1.0×, with the aperture being manually adjustable (F4,5–F22). In order to maintain the depth of focus within the range of the glare points, it was found that aperture values of approximately F6–F8 were optimal. The trigger signal of the dispenser is connected to the camera. Consequently, by setting the shutter delay in accordance with the desired image acquisition time, the camera can be configured to capture the precise moment when a droplet passes by. Adjustments to align the droplet position in the image in the z-direction (Figure 2) can be made by changing the trigger delay time. The alignment of the droplets in the direction of the camera axis (y-axis in Figure 2) is achieved by adjusting the focal length of the lens. Once the system has been set up, it is unnecessary to make any adjustments perpendicular to the camera axis (x-axis in Figure 2). As a single droplet is ejected per stroke, the number of images captured represents the number of droplets dispensed. Furthermore, a second camera system (Basler daA3840-45um) was employed to capture images of a microscope slide on which the droplets were dispensed. The reference camera permits the number of beads or spheroids to be counted and the number of particles on the microscope slide to be compared with the number of particles detected in the images of free-falling droplets.

### 2.3. Illumination Concepts

#### 2.3.1. Multiple Colored LED Illumination

**Figure 3:**
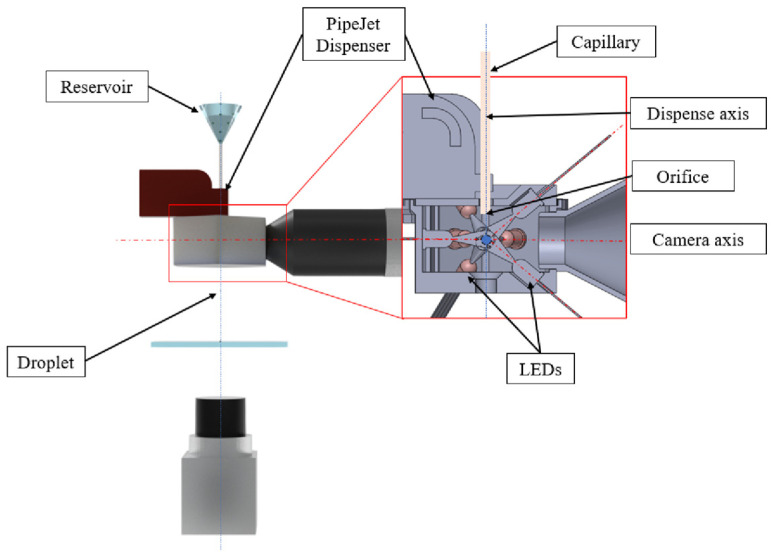
The housing, which includes the LEDs and adapts the camera as well as the dispenser for the detection of the droplets.

**Figure 4:**
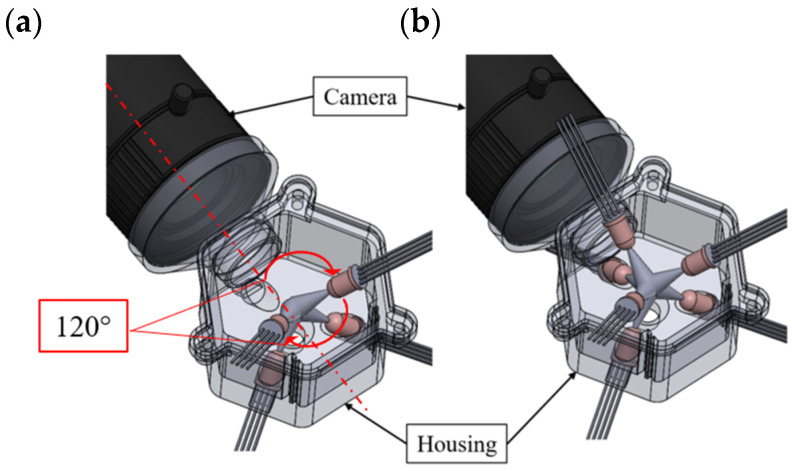
(**a**) Setup with four-LED illumination posterior. (**b**) Setup with six-LED posterior and anterior illumination.

Droplets with a volume of 30 nl were continuously dispensed in the test setup described in the previous section. In the first step, we used LED light with either 405 nm (violet), 465 nm (blue), or 635 nm (red) wavelengths for the illumination of ejected droplets. The LEDs were arranged on the top and bottom of a specially designed housing at an angle of 40° towards the optical axis of the CMOS camera and at an angle of 60° around the dispense axis, as shown in Figure 3 and Figure 4. At an angle of 60° the LEDs were equally distributed around the dispense axis. An angle of 40° vertical to the dispense axis was chosen to adapt to the 3D-printing manufacturing process of the housing. Furthermore, it ensured that the axis along which the light emitted by the LED propagates intersected the camera axis at the point where the droplet dispensing axis intersected the camera axis. To be able to capture the droplets always in the same position, the camera captured images triggered by the dispensing signal of the PipeJet nanodispenser.

The light setup depicted in Figure 3 shows the experimental LED arrangement. The LEDs were aligned posterior to the camera. Depending on the experiment, distinct LEDs can be used to set up the LED arrangements with four (Figure 4a) and six LEDs (Figure 4b).

#### 2.3.2. Diffuse Anterior Illumination with White Light

**Figure 5:**
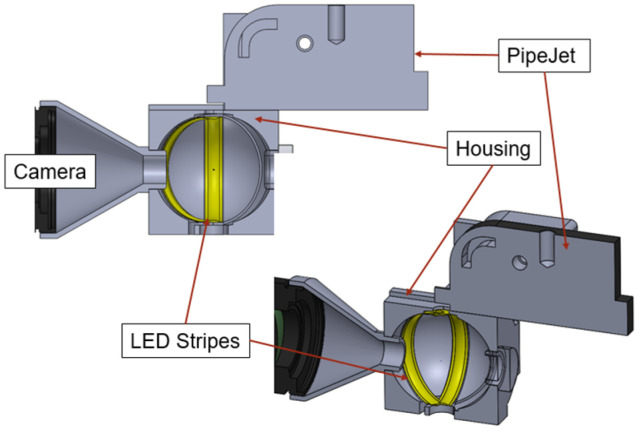
Four LED stripes arranged in a half-spherical configuration around the droplets. The LED stripes illuminate the droplets in an anterior direction with respect to the camera.

Rather than using direct illumination of the droplets, LED stripes (24 V, 3 W, 100 lumens) were used. The white light emitted by the LEDs scattered diffusely due to the coating on the stripes. The walls of the housing to which the stripes were attached in this concept were spherical in shape, as shown in Figure 5. The color of the housing was chosen to be white to enhance the reflection and diffuse scattering of the light emitted by the LED stripes. Thus, light penetrated the droplets from all directions and overcame the focusing behavior of the droplets. The setup was mounted to the experimental setup described in Section 2.2.

### 2.4. Image Acquisition

For testing the different illumination concepts the following particles were used for the experiments:

- Blue-colored polyethylene (PE) beads with a diameter range of 90–106 µm (Cospheric BLPMS-1.08 polyethylene microspheres, Cospheric LLC, Somis, CA, USA)
- Blue-colored PE beads with a diameter range of 45–53 µm (Cospheric BLPMS-1.08 polyethylene microspheres, Cospheric LLC, Somis, CA, USA)
- Transparent PE beads with a diameter of 75 µm (Polysciences Inc. Polybead, polyethylene, 75 µm, Cospheric LLC, Somis, CA, USA)
- MCF-7 spheroids that were cultivated according to Section 2.1 within a diameter range of 80–130 µm. Spheroids were dispensed by the test setup on the day of harvesting.

A minimum of three image series with 200 images in each series were captured for each particle type and illumination concept. The dispensed number of particles per run varied between 6 and 30 particles; no other dispensed droplets contained any particle.

To be able to compare different image analysis algorithms to each other, all algorithms evaluated the same set of images. Moreover, the number of dispensed particles was determined for each set by the reference camera system, which imaged the microscope slide. Finally, the droplets which were identified to contain particles by the algorithms could be compared to the actual number of dispensed particles using Equation (1).

(1) $$\;A=\frac{Number\;of\;particles\;detected\;in\;free\text{-}falling\;droplet\;images}{Actual\;number\;of\;particles\;on\;microscope\;slide}\times 100\%$$

### 2.5. Image Evaluation

Different computer-based image analysis algorithms (white pixel count, contour count, droplet image similarity) were tested and compared with regard to their ability to detect particles in free-falling droplets. The applied and tested algorithms are briefly described in the following sections.

#### 2.5.1. White Pixel Count (WPC)

**Figure 6:**
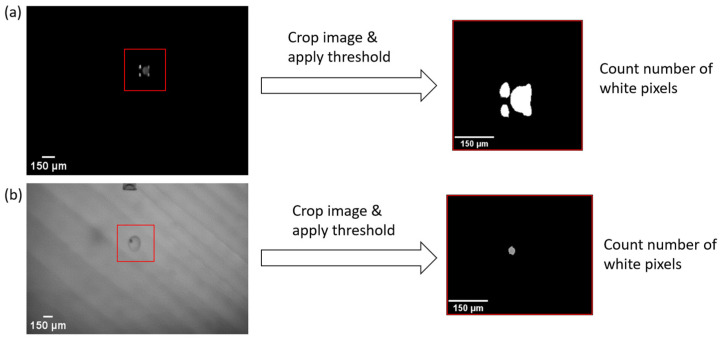
(**a**) Glare point image of a free-falling droplet with included bead particle (90–106 μm). **Left side**: raw, unprocessed image; **right side**: same image after cropping and applying Otsu threshold. (**b**) Diffuse illuminated image of a free-falling droplet with included bead particle (45–53 μm). **Left side**: raw, unprocessed image. **Right side**: image after cropping and applying a hard threshold.

Calculating the mean value of pixel values of an image is one possible way of determining differences in images dependent on droplet inclusion. The process based on captured images is depicted in Figure 6.

The images were first cropped to a size of 200 × 200 pixels. This is the image size that is well suited for fast image processing and is suited, as well, to the size of the droplets. In the second step, an Otsu thresholding was applied to convert the image to a binary image. Then, the number of white pixels was counted and divided by the total number of pixels of the image, which resulted in the WPC score (Equation (2)).

For this approach, it is assumed that images containing particles should have a higher or lower number of white pixels because the glare points change in their appearance or appear or disappear, leading to an increase or decrease in the number of white pixels. Consequently, the mean pixel value also changes. The same holds true for the images captured with diffuse illumination. A cut-off value is defined by calculating the average mean value of ten empty droplet images. A margin of 15% of that value is added to define the particle detection cut-off. All images of droplets above that cut-off are considered to contain a particle. This method is called the white pixel count (WPC).

(2) $$WPC=\frac{Number\;white\;pixels}{Total\;number\;of\;pixels}$$

#### 2.5.2. Contour Count (CC)

In the case of the illumination concept with multiple, colored LEDs, multiple glare points are projected onto the surface of the droplets. Particles within the droplets influence the appearance of these glare points. Depending on the size of the particles, it is assumed that some glare points will disappear, or new glare points appear.

The evaluation of the contour count is based on this assumption and calculates the number of individual glare points on the surface. A more advanced version also considers the size of the surface which is enclosed by these contours and compares it to a reference droplet.

**Figure 7:**
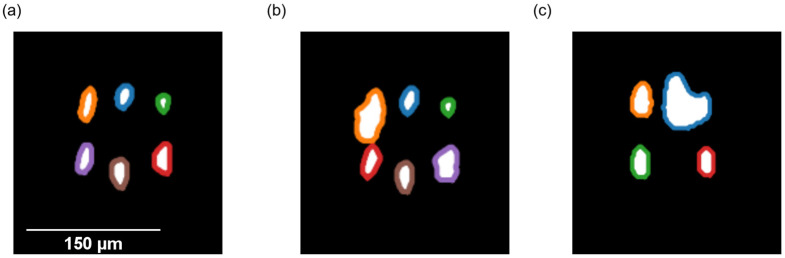
(**a**) Image of an empty droplet with six contours that were drawn around the glare points by the algorithm. (**b**) Image of a droplet containing a bead particle. The area enclosed by the orange contour increases in size. (**c**) Image of a droplet containing a bead particle. The brown glare point disappeared whereas the blue one increased in size.

As a first step, the captured images were therefore cropped to a size of 200 × 200 pixels, and the Otsu threshold was applied, as mentioned in the previous section. To reduce the number of single black or white pixels, a gaussian filter was used for further processing of the images. Contour detection was performed with the Python library skimage [20]. Figure 7 shows sample images with glare points which were detected by the described computer-based image processing.

To determine the surface area of the individual contours, the OpenCV (OpenCV—Contours in OpenCV: https://docs.opencv.org/3.4/d3/d05/tutorial_py_table_of_contents_contours.html accessed on 31 March 2024) contours feature was used and implemented into the code. The values of the individual point areas were then compared to the areas for the same contours on the droplets which were imaged before. Increases or decreases of more than 30% of the size which was determined previously were considered as a detection.

#### 2.5.3. Droplet Image Similarity (DIS)

**Figure 8:**
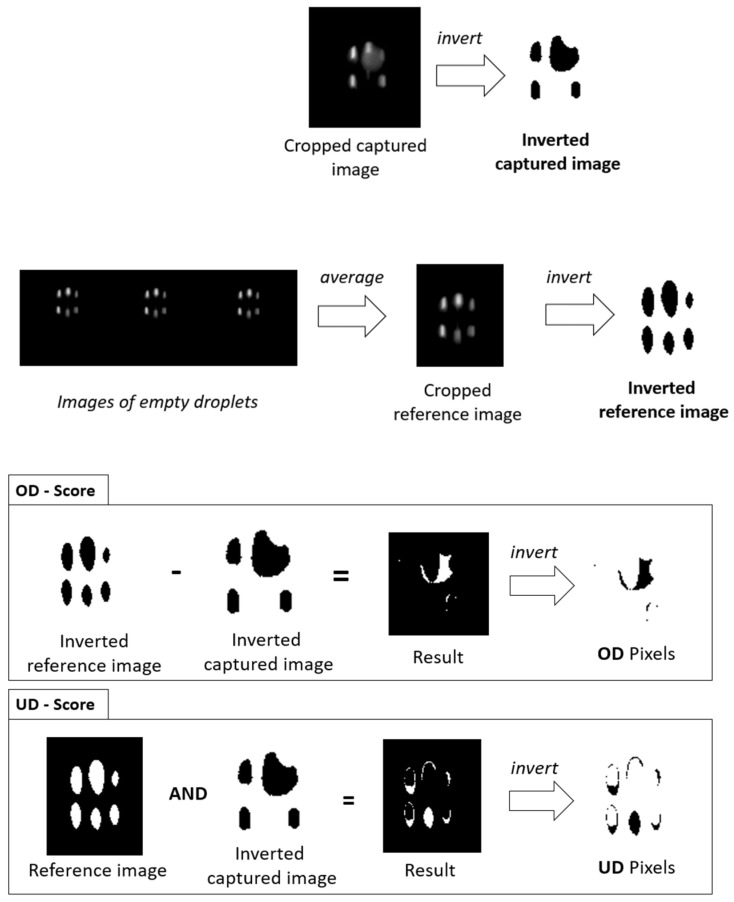
Image processing for calculating the droplet image similarity coefficient. First, images from empty droplets are used to calculate a reference image which can be compared to the captured images. This comparison is based on the OD and UD score. The process of calculating the OD score and UD score by mathematical operations carried out on the matrixes of the images is depicted in the lower half of the image. Afterwards, the number of black pixels in the resulting images are counted. If the number is higher than a pre-defined cut-off, the droplet image can be considered as a detection.

Another method to determine differences in images of droplets with or without an enclosed particle is the calculation of droplet image similarity (DIS), which is determined based on the printing accuracy model developed by Fritz Koch et al., 2023 [21]. It consists of three scores. One counts the number of pixels that change color from black to white in an image compared to a reference image (OD). The second score counts the number of pixels that are missing (change color from white to black) compared to a reference image (UD). Both scores combined lead to the third score, which indicates the similarity of the captured image to the reference image (DIS). The reference image in the case of the droplet images was calculated using the mean of 20 images of empty droplets. The equations of the scores, as well as the determination of the scores by image processing, are shown in Figure 8. The binarization of the images was performed by applying the Otsu threshold to the images. To compensate for positional discrepancies between the droplets in the captured images, a template-matching algorithm was employed. This algorithm convolved a reference image with the captured images in two dimensions, thereby identifying the coordinates where the two images exhibited the greatest degree of alignment.

The calculation of the three scores allows for the detection of a deviation from a defined cut-off to be counted as soon as two of the three scores deviate simultaneously.

(3) $$UD=\frac{missing\;pixels}{reference\;pixel}\times 100\%$$

(4) $$OD=\frac{missing\;pixels}{reference\;pixel}\times 100\%$$

(5) $$DIS=100\%-\frac{UD+OD}{2}$$

## 3. Results and Discussion

### 3.1. Wavelength and Number of Light Sources

**Figure 9:**
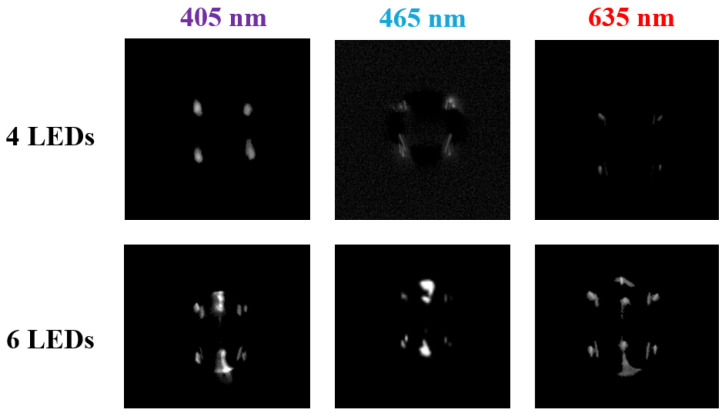
Empty droplets illuminated by four and six LEDs in the arrangement as shown in Figure 2 with different wavelengths. The size of droplets varied in the range of 80 μm up to 130 μm.

The initial investigation focused on the characteristic glare points of empty water droplets, employing anterior and posterior light modalities. The droplets were illuminated by evenly distributed LEDs around the dispense axis, resulting in bright glare points, using either anterior or anterior-posterior illumination for all wavelengths (see Figure 9). The differences between illumination concepts are primarily reflected in the number of glare points on the droplet surface and the varying brightness levels of these points, which depend on the wavelength and number of light sources used. The greater number of glare points on droplets illuminated by six LEDs suggested that the information gained from these points might be more extensive than that gained from droplets illuminated by four LEDs. Consequently, further experiments were only conducted with the six LED arrangement.

For subsequent experiments, MCF-7 spheroids with diameters ranging from 80 to 130 μm were cultivated, harvested, and resuspended in phosphate-buffered saline (PBS) for dispensing.

**Figure 10:**
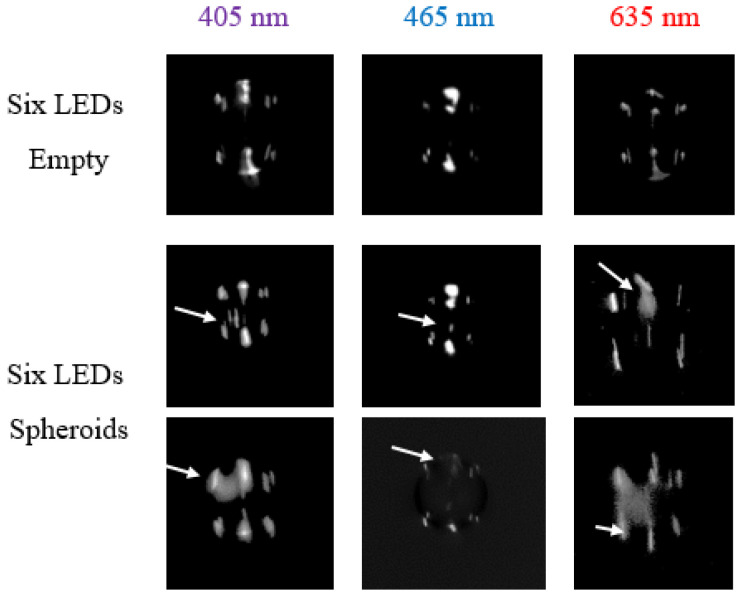
Series of captured images of droplets with different wavelengths of illuminating light. The change of glare points, which indicates a detection of spheroids, is marked with arrows. The droplet size was in the range of 80 μm up to 130 μm.

Five image series, each containing 200 images, were captured using the spheroid PBS suspension for the tests. This process was repeated for each wavelength. The droplets were dispensed onto a microscope slide, and the number of dispensed spheroids was counted under the microscope after each run. An experienced researcher manually evaluated the images and counted the number of images with potential detections. Figure 10 shows sample images of droplets. New bright points or large points with lower intensity appear as soon as a spheroid is encapsulated in the droplet. This effect is most pronounced for a wavelength of 405 nm. The reason for this effect can be seen in the dispersion effect caused by the different wavelengths. In the case of 405 nm, the light is refracted to a higher degree than light with a wavelength of 635 nm [22]. Consequently, the 405 nm light propagates on different path within the droplet compared to light with a higher wavelength. Hereby, the light hits the backside of the droplet at a sharper angle. This causes a reflection at a sharper angle with respect to the center axis of the droplet (see Figure 1), resulting in a higher intensity on the camera sensor as more light is guided towards the lens setup. This, in turn, produces brighter images of the glare points. As soon as a particle is within the path of light propagation within the droplet, the refraction caused by this particle is, furthermore, stronger due to the different refraction angle, resulting in more pronounced changes in the appearance of the glare point. In accordance with Snell’s law, which describes the change of the direction of the incident light when transferring from one medium to another, depending on the refractive index of the media, the same effect can be achieved by different droplet media, provided that the refractive indices of the droplet media differ to a significant extent.

In the next step, the number of images in which the researcher detected a spheroid within the droplet and the number of spheroids counted using the microscope were compared to calculate the manual detection accuracy according to Equation (1).

**Figure 11:**
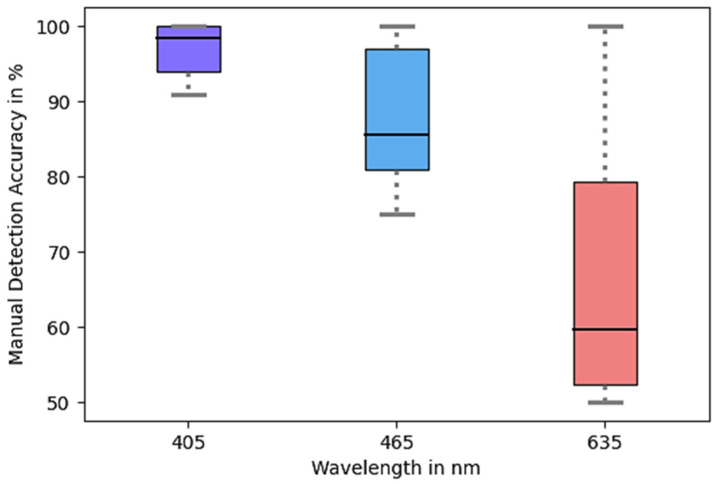
Spheroid (80 μm up to 130 μm) detection accuracy of the different wavelengths. The droplets were illuminated by six LEDs which could emit the three different wavelengths. Each wavelength was tested five times with 200 images each time.

The detection accuracy of the version with violet light was more constant and on average higher (93.8%) than with red (59.2%) and blue (84.3%) light. This corroborates the observation that light of a shorter wavelength is deflected more strongly by particles with shorter wavelengths, resulting in greater alterations in the size and intensity of the glare point. The distribution of the accuracies can be seen in Figure 11.

Based on the manual evaluation, it was decided to proceed with the illumination using six 405 nm LEDs for further experiments towards an automated process. Based on the best accuracies, this setup demonstrated the most promising results in terms of glare point clarity and difference between an empty droplet and a droplet containing a spheroid.

### 3.2. Code-Assisted Evaluation

Next, we evaluated the effect of different particle types, as mentioned in the Section 2, on detection accuracy.

For each particle type, we took three series of 200 images each. These images were later analyzed on a separate PC using either the white pixel count, contour count, or droplet similarity algorithm. After each run, we counted the total number of particles ejected using the reference camera pointing at the microscope slide on which the droplets were ejected.

Table 1 displays the detection accuracies achieved by the three considered detection algorithms based on images captured with directed illumination using a six-LED arrangement and violet light.

All three algorithms showed a strong performance on particles larger than 53 μm. For particles smaller than 45 μm, glare point changes became less significant relative to the size of the glare points and were indistinguishable from noise effects. Slight position shifts of droplets within the captured images, for example due to trigger jitter, can cause noise. This leads to a constant, slight change in the glare point position due to the changing angle of incidence.

The droplet image similarity algorithm is highly dependent on identical images, as they are subtracted from one another. Minor variations in droplet appearance and, consequently, glare point shape, can result in increased score values that are comparable to those caused by small particle-induced changes in glare point size. It was not possible to distinguish these errors from actual detections of particles that were in a size range of 45–53 μm. Nevertheless, a comparison of the captured images with a reference image demonstrated significant potential for all other tested particles, particularly spheroids.

The CC algorithm is less prone to errors caused by droplet position changes, as it only counts the number of glare points. However, it may struggle to detect small particles only by the size change of a single glare point, as the glare point only changes in size instead of forming a new one. Additional parameters, such as the area of individual glare points, have shown potential to further increase detection accuracy.

In terms of the time required for image processing to evaluate the presence of particles within droplets, the WPC algorithm can deliver a result within 0.06 s per image. Due to the higher amount of processing steps required for the CC and DIS algorithms, processing times are four to five times higher. In order to achieve a real-time detection, it is necessary to further improve the programming language and the hardware configuration of the PC used to process the image.

**Figure 12:**
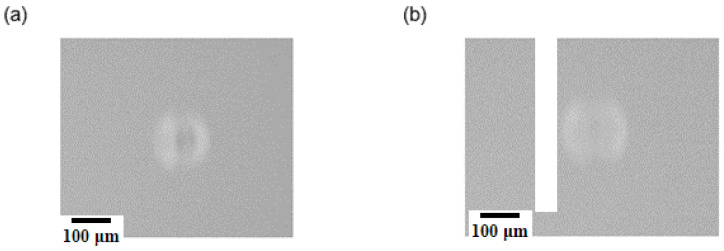
(**a**) Empty droplet image with diffuse anterior illumination. (**b**) Droplet containing a spheroid imaged with diffuse anterior illumination. A comparison of the two images shows no significant difference, which makes a detection of spheroids impossible.

**Figure 13:**
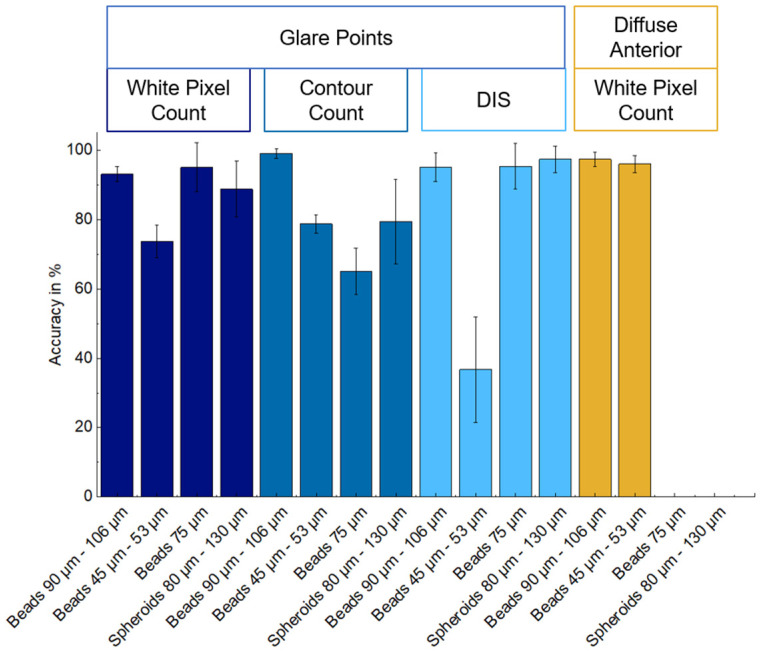
Comparison of the overall achieved detection accuracies of the different evaluation algorithms and illumination concepts. Each bar depicts the mean accuracy calculated from three runs with 200 evaluated images in each run. The error bars show the standard deviation.

Table 2 presents the results achieved by the white pixel count algorithm based on images captured with the diffuse illumination concept. The algorithm demonstrated an accuracy of greater than 95% for blue-colored beads with a size range of 45 μm to 106 μm. However, the algorithm was unable to detect spheroids and transparent beads, as illustrated in Figure 12. The diffuse illumination resulted in diffuse light reflections indistinguishable from spheroids and transparent particles. Nevertheless, the evaluation algorithms indicated that the accuracy for light-absorbing colored particles was higher than that achieved with the directed light concept. Figure 13 provides a side-by-side comparison of the achieved accuracies. The processing of glare point images, in particular with the DIS algorithm, is most effective for transparent particles and spheroids. This is due to the fact that pronounced changes in the glare point appearance can be easily distinguished from empty droplet glare point images.

The impact of the aforementioned errors is most pronounced for small opaque particles, as evidenced by the weakness of the algorithm that employs the comparison of images (DIS). The diffuse light setting is the most reliable for the detection of small opaque particles.

A satisfactory compromise is the evaluation of images by the WPC and CC algorithms, which exhibit lower but still high accuracies for the complete range of tested particles. Furthermore, the CC algorithm has the potential to be enhanced by calculating the area of individual contours and comparing them to empty droplet images or previously captured images. It is anticipated that further enhancements in the detection accuracy for small particles will be achieved through a reduction in the size of the glare points. Alternatively, it would be more effective to project more of these smaller, distinct glare points onto the droplets. The alterations compared to the characteristics of individual glare points become more pronounced, thereby facilitating their identification.

The differentiation of the tested particles was not within the scope of the tested concepts. The captured images nevertheless demonstrated a tendency for the glare points to exhibit a different appearance depending on particle size and type. This could be utilized in the future to sort different sizes or types of particles. A larger particle size, for example, results in a greater increase in the glare point surface. Changing refractive indices between particles and spheroids causes the same effects.

## 4. Conclusions

The concept study presented here demonstrates the possibility of determining the presence of a particle within a free-falling droplet using glare points projected onto its surface or diffuse illumination. This approach offers greater flexibility in the selection of materials for dispensing nozzles, while enabling the detection of particles or cellular aggregates that have left the dispenser without the need for special camera equipment such as high-speed cameras. Moreover, there is no need for fluorescent labels, which can influence the results of biological experiments, to detect these particles.

For glare point projection, six LED lights with a wavelength of 405 nm were arranged anteriorly and posteriorly towards the droplet. Particles within the droplets could be detected by means of characteristic glare point changes with respect to empty droplets. Algorithms were proposed and tested for image analysis. The computer-based detection accuracy achieved was comparable to that of manual image evaluation. For particles equal to or larger than 75 μm, high detection accuracies of >95% were achieved. However, the accuracy decreased for smaller particles. The reason for this can be observed in the comparison of glare points to the particle sizes. The introduction of smaller glare points and their respective changes, in addition to a higher number of glare points projected onto the droplets, represents a potential avenue for enhancement, with the objective of improving the detection of smaller particles. The diffuse white light concept accounts for the lens-like behavior of spherical droplets by uniform illumination from all directions, thus reducing dark drop edge areas. This allowed high detection accuracies of 96%, even for small opaque particles with a size of 45 μm. Nevertheless, with this concept, spheroids and transparent particles cannot be detected.

This study showed that droplet imaging techniques can enhance the quality of the Drop-on-Demand bioprinting process by accurately determining the dispensed quantity of particles. This method is independent of dispense head design and dispense parameters, as it detects particles within the already dispensed droplets. Enabling more freedom in the design of the dispense head allows for the easy exchange of nozzles or capillaries without the need to adapt the system for particle detection. This might be especially useful in terms of automated workflows, where printheads need to be changed according to the different print materials.

The combination of the two illumination concepts and their respective evaluation algorithms is anticipated to enhance particle detection for a wide range of particles, as each concept demonstrates strengths for different particle types and sizes. To achieve this, both illumination concepts could be integrated into a single housing. Filters can be applied to the imaging system to capture two images of the same droplet simultaneously and evaluate them using either WPC or DIS algorithms. It would be advantageous to consider approaches to making the entire detection system more compact in size and to reduce the free-falling path of the droplets in future research. One potential solution is the implementation of optical fibers instead of LEDs, which could help to reduce the size of the module and enhance the glare point appearance due to more focused light propagation towards the droplet. Nevertheless, this technique can assist in enhancing the quality of material jetting printing processes and, especially, the Drop-on-Demand bioprinting process by enabling a more precise determination of the actual dispensed amounts of particles, independent of the printhead material and without the necessity for fluorescent labels. Consequently, the present concept enhances the reliability of drug screening research outcomes on printed models.

## Figures and Tables

**Table 1:** Achieved accuracy (A) of white pixel count (WPC), contour count (CC), and droplet image similarity (DIS) for direct illumination compared to each other based on the same images.

Particle	A—WPC [%]	A—CC [%]	A—DIS [%]
Beads 90–106 μm	93.0 ± 2.2	99.0 ± 1.4	95.0 ± 4.1
Beads 45–53 μm	73.7 ± 4.7	78.7 ± 2.6	36.7 ± 15.3
Beads 75 μm	95.0 ± 7.1	65.0 ± 6.7	95.3 ± 6.1
Spheroids 80–130 μm	88.7 ± 8.1	79.3 ± 12.2	97.3 ± 3.8

**Table 2:** Accuracy of white pixel count (WPC) for diffuse illumination.

Particle	A—WPC [%]
Beads 90–106 μm	97.3 ± 2.1
Beads 45–53 μm	96 ± 2.5
Beads 75 μm	-
Spheroids 80–130 μm	-

## Data Availability

The data presented in this study are available on request from the corresponding author. The data are not publicly available for privacy reasons.

## References

[1] Gutzweiler L., Kartmann S., Troendle K., Benning L., Finkenzeller G., Zengerle R., Koltay P., Stark G.B., Zimmermann S. (2017). Large scale production and controlled deposition of single HUVEC spheroids for bioprinting applications. Biofabrication.

[2] Dornhof J., Zieger V., Kieninger J., Frejek D., Zengerle R., Urban G.A., Kartmann S., Weltin A. (2022). Bioprinting-Based Automated Deposition of Single Cancer Cell Spheroids into Oxygen Sensor Microelectrode Wells. Lab A Chip.

[3] Zieger V., Woehr E., Zimmermann S., Frejek D., Koltay P., Zengerle R., Kartmann S. (2024). Automated Nanodroplet Dispensing for Large-Scale Spheroid Generation via Hanging Drop and Parallelized Lossless Spheroid Harvesting. Micromachines.

[4] Zieger V., Frejek D., Zimmermann S., Miotto G.A.A., Koltay P., Zengerle R., Kartmann S. (2024). Towards Automation in 3D Cell Culture: Selective and Gentle High-Throughput Handling of Spheroids and Organoids via Novel Pick-Flow-Drop Principle. Adv. Healthc. Mater..

[5] Dababneh A.B., Ozbolat I.T. (2014). Bioprinting Technology: A Current State-of-the-Art Review. J. Manuf. Sci. Eng..

[6] Mironov V., Visconti R.P., Kasyanov V., Forgacs G., Drake C.J., Markwald R.R. (2009). Organ Printing: Tissue Spheroids as Building Blocks. Biomaterials.

[7] Parent C., Melayil K.R., Zhou Y., Aubert V., Surdez D., Delattre O., Wilhelm C., Viovy J.-L. (2023). Simple Droplet Microfluidics Platform for Drug Screening on Cancer Spheroids. Lab A Chip.

[8] Lee J.M., Sing S.L., Zhou M., Yeong W.Y. (2018). 3D bioprinting processes: A perspective on classification and terminology. Int. J. Bioprinting.

[9] Tropea C. (2011). Optical Particle Characterization in Flows. Annu. Rev. Fluid Mech..

[10] Li L., Tropea C. (2021). Measurement of the colloidal particle concentration and size within a drop using the time-shift technique. J. Quant. Spectrosc. Radiat. Transf..

[11] Schröder G. (2014). Technische Optik. Grundlagen und Anwendungen.

[12] Nagelberg S., Zarzar L.D., Nicolas N., Subramanian K., Kalow J.A., Sresht V., Blankschtein D., Barbastathis G., Kreysing M., Swager T.M. (2017). Reconfigurable and Responsive Droplet-Based Compound Micro-Lenses. Nat. Commun..

[13] Huang X., Ng W.L., Yeong W.Y. (2023). Predicting the Number of Printed Cells during Inkjet-Based Bioprinting Process Based on Droplet Velocity Profile Using Machine Learning Approaches. J. Intell. Manuf..

[14] Heinisch C. (2008). Optische Messtechnik für Umströmte Tropfen in einer Neuen Elektrodynamischen Falle. Technische Universität Darmstadt. https://tuprints.ulb.tu-darmstadt.de/1199/1/Heinisch2008_Optische_Messtechnik_fuer_umstroemte_Tropfen_in_einer_neuen_elektrodynamischen_Falle.pdf.

[15] Kreibig U. (2008). Hundert Jahre Mie-Theorie. Optische Eigenschaften von Nanopartikeln. Phys. Unserer Zeit.

[16] Ray-Optics-Simulation. https://phydemo.app/ray-optics/.

[17] Costa E.C., Silva D.N., Moreira A.F., Correia I.J. (2019). Optical Clearing Methods: An Overview of the Techniques Used for the Imaging of 3D Spheroids. Biotechnol. Bioeng..

[18] Zhang W., Li C., Baguley B.C., Zhou F., Zhou W., Shaw J.P., Wang Z., Wu Z., Liu J. (2016). Optimization of the Formation of Embedded Multicellular Spheroids of MCF-7 Cells: How to Reliably Produce a Biomimetic 3D Model. Anal. Biochem..

[19] Streule W., Lindemann T., Birkle G., Zengerle R., Koltay P. (2004). PipeJet: A Simple Disposable Dispenser for the Nano- and Microliter Range. JALA J. Assoc. Lab. Autom..

[20] Lorensen W.E., Cline H.E. Marching Cubes: A High Resolution 3D Surface Construction Algorithm. Proceedings of the 14th Annual Conference on Computer Graphics and Interactive Techniques.

[21] Koch F. (2023). Quantitative Assessment of Generic Processes to Enable 3D-Bioprinting of Artificial Tissue. Ph.D. Thesis.

[22] Broemser P. (1925). Farbenzerstreuung bei der Brechung des Lichts, Spektrum. Einführung in Die Physik.

